# Play along: effects of music and social interaction on word learning

**DOI:** 10.3389/fpsyg.2015.01316

**Published:** 2015-09-01

**Authors:** Laura Verga, Emmanuel Bigand, Sonja A. Kotz

**Affiliations:** ^1^Department of Neuropsychology, Research Group Subcortical Contributions to Comprehension, Max Planck Institute for Human Cognitive and Brain SciencesLeipzig, Germany; ^2^Movement to Health Laboratory (M2H), EuroMov – Montpellier-1 UniversityMontpellier, France; ^3^Laboratoire d’Etude de l’Apprentissage et du Développement, Department of Psychology, University of BurgundyDijon, France; ^4^School of Psychological Sciences, The University of ManchesterManchester, UK

**Keywords:** music, social interaction, word learning, temporal coordination, contextual learning

## Abstract

Learning new words is an increasingly common necessity in everyday life. External factors, among which music and social interaction are particularly debated, are claimed to facilitate this task. Due to their influence on the learner’s temporal behavior, these stimuli are able to drive the learner’s attention to the correct referent of new words at the correct point in time. However, do music and social interaction impact learning behavior in the same way? The current study aims to answer this question. Native German speakers (*N* = 80) were requested to learn new words (pseudo-words) during a contextual learning game. This learning task was performed alone with a computer or with a partner, with or without music. Results showed that music and social interaction had a different impact on the learner’s behavior: Participants tended to temporally coordinate their behavior more with a partner than with music, and in both cases more than with a computer. However, when both music and social interaction were present, this temporal coordination was hindered. These results suggest that while music and social interaction do influence participants’ learning behavior, they have a different impact. Moreover, impaired behavior when both music and a partner are present suggests that different mechanisms are employed to coordinate with the two types of stimuli. Whether one or the other approach is more efficient for word learning, however, is a question still requiring further investigation, as no differences were observed between conditions in a retrieval phase, which took place immediately after the learning session. This study contributes to the literature on word learning in adults by investigating two possible facilitating factors, and has important implications for situations such as music therapy, in which music and social interaction are present at the same time.

## Introduction

In an increasingly multicultural world, even adult speakers often face the necessity to acquire a foreign language starting from its building blocks: words. New words are frequently encountered in everyday life, and the first step to learning them is to understand what they mean. However, possible meanings for a new verbal label are countless. How does the learner identify the correct one? Research in second language learning has identified several factors that may facilitate learners in their effort to acquire new vocabulary, among which music and social interaction stand out as particularly important, yet their role is still debated.

The idea that music may boost language functions has fascinated the scientific community for quite some time (Schellenberg, 2003), with particularly convincing evidence coming from clinical studies (Hillecke et al., 2005; Thompson et al., 2005; de l’ Etoile, 2010; Simmons-Stern et al., 2010; Thaut, 2010; Hurkmans et al., 2011; Altenmüller and Schlaug, 2013). Similarly, in healthy populations several studies report a positive effect of music on the encoding and decoding of verbal material, with music being used either as a background (De Groot, 2006; Ferreri et al., 2014, 2013), as a contrast for sung and spoken material (Rainey and Larsen, 2002; Ludke et al., 2014), or as a form of long-term training (Kilgour et al., 2000; Ho et al., 2003). The question remains open, however, as to which specific aspects of music impact learning. It has been proposed that the boosting effect of music may depend on different mechanisms (for example, temporal scaffolding/attention, emotion/reward and arousal/mood), recruited by progressively higher levels of musical complexity (Ferreri and Verga, under review). In particular, this account suggests that simple musical stimuli aligned with verbal material may significantly potentiate learning by providing a temporal structure, in which temporal regularities orient participants’ attention to the verbal information to be encoded (Jones and Boltz, 1989; Thaut et al., 2005; Schön et al., 2008; Francois and Schön, 2010); in the case of vocabulary learning, this information is represented by new words and their respective referents. By facilitating predictions of “what is coming next” (Tillmann et al., 2003; Collins et al., 2014; Mathias et al., 2014), the temporal regularities conveyed by music also induce temporal coordination^1^. Indeed, a tight link between music and coordinated motor behavior emerges very early on in life (for example see Phillips-Silver and Trainor, 2005) and continues throughout the entire lifespan, as demonstrated by the fact that listeners often “tap their feet or nod along to the beat of a tune” (Chen et al., 2008; see also Loehr et al., 2011; Repp and Su, 2013). Importantly, this form of auditory-motor synchronization to music has been shown to further improve attentional processing, by facilitating the temporal encoding of the stimuli (Schmidt-Kassow et al., 2013).

Interestingly, similar mechanisms (that is, attention orienting and temporal coordination) have been proposed to explain the facilitating effect of social interaction on word learning in children, for whom the presence of another person is a *sine qua non*-condition to build up new vocabulary (Kuhl et al., 2003; Kuhl, 2007). In these asymmetric learning settings the role of the more experienced person is to guide the learner’s attention toward the correct referent for a new word, thus strongly reducing the number of possible referents (Csibra and Gergely, 2009; Hirotani et al., 2009). For this facilitation to occur, temporal coordination between the learner and the social partner is required to triangulate attention toward the target referent at the correct point in time (Gogate et al., 2000; Rolf et al., 2009; Rader and Zukow-Goldring, 2012). Support for this claim comes from evidence that children learn significantly more new words when they are able to reach a good temporal coordination with their caregiver (Pereira et al., 2008). However, as social verbal learning in adults has not been the focus of research until recently (Jeong et al., 2010, 2011; Verga and Kotz, 2013), the impact of a partner on second language acquisition still remains an open question. Similarly to children, coordination with a more experienced partner may create a sort of “multi-modal rhythm” capable of facilitating the allocation of attention and the binding of information required for learning (that is, the correct referent and its new verbal label; Lagarde and Kelso, 2006; Rolf et al., 2009). While the emergence of spontaneous temporal coordination during interactive social situations is frequently and reliably reported in literature on joint action (for example Richardson et al., 2007; Yun et al., 2012), its impact on word learning has not yet been investigated.

The evidence reported so far suggests that common properties in music and social interaction – such as the establishment of a temporal structure – may boost word learning by facilitating the allocation of attention and the emergence of spontaneous temporal coordination. Importantly, however, these are not the only commonalities between music and social interaction that justify a comparison between the two stimuli: indeed, they are both rich, complex stimuli that are pleasurable and enjoyable (Blood et al., 1999; Hari and Kujala, 2009) which are often concurrently present in a number of contexts (for example, musical performance, music therapy). Nevertheless, an important distinction needs to be made: Listening to music has a unidirectional influence, in the sense that the listener coordinates with the music, but not vice-versa (Repp and Keller, 2008). Instead, social interaction elicits a bidirectional influence between partners, who tend to reciprocally modify their behavior (Richardson et al., 2007; Yun et al., 2012). In this scenario, predictions about what is coming next need to be constantly updated in order to allow the adaptation of one’s own behavior, an ability critically dependent on the typically human skill to infer the other person’s intentions (Frith and Frith, 2006, 2012). Whether this difference influences the way temporal coordination is achieved represented the topic of a recent study by Demos et al. (2012). In their experiment, these authors evaluated participants’ coordination with music or a partner, while seated in rocking chairs, and observed that spontaneous coordination emerged with music as well as with a partner. However, coordination with music was weaker than with a partner. Further, when both music and the partner were present, they competed as sources of attraction, resulting in a weaker coordination. The authors interpret these results by proposing that coordination with music differs from coordination with a partner because people interacting together behave as coupled oscillators (Wilson and Wilson, 2005; Dumas et al., 2010; Demos et al., 2012). At the neural level, this behavior reflects the activity of populations of neurons in the member of the dyad which become synchronized in their oscillating firing pattern (Dumas et al., 2010, 2011; Cui et al., 2012; Hasson et al., 2012), and in turn, this coupling is reflected in temporal coordination emerging at the behavioral level (Richardson et al., 2007; Pereira et al., 2008; Yun et al., 2012). From a psychological standpoint, these phenomena create a “common ground” between partners, facilitating the transmission of information (Csibra and Gergely, 2009). While this psychological state has been deemed pivotal for children to determine the adult’s referent of a new word (Tomasello, 2000), whether adult learners may also benefit from this “shared ground” is still an open question (Pickering and Garrod, 2004; Jeong et al., 2010; Stephens et al., 2010; Verga and Kotz, 2013). On the one hand, the presence of a knowledgeable partner may help to reduce the number of possible referents for a new word; on the other hand, adults do posses – compared to infants – more refined cognitive abilities, which may be sufficient for acquiring new words. In the first case, the role of the social partner may be to provide a temporal structure able to drive participants’ attention toward the verbal information to be encoded; in this case, the source of information (whether a human or not) should be irrelevant. Conversely, if the establishment of a “common ground” – partially reflected by temporal coordination between the partners – is as important in adult learners as it is in infants, then social interaction should provide an advantage when compared to other forms of temporally structured stimuli, such as music. In other words, this corresponds to the question of whether it is necessary for this temporal structure to be conveyed by *someone*, or if it is enough for it to be conveyed by *something*.

In the current study, our aim was to answer this question by implementing a social/non-social contextual learning task that could be performed either with or without music. In this task, a participant interacts with an experimenter on a checkerboard containing images depicting either nouns or verbs; their common goal is to identify three images, which combined together create a plausible sentence in the form subject-verb-object. When they reach their goal, the name of the sentence object in a new language is presented to the participant. While the game approach is typical for social interaction studies (e.g., De Ruiter et al., 2007), the current word learning game represents a novel paradigm for language learning studies.

Based on the literature reviewed above, we expected participants to achieve better temporal coordination with a social partner (Richardson et al., 2007; Yun et al., 2012) and with music (Repp and Keller, 2008; Demos et al., 2012) when compared to a computer, but hindered when both music and social interaction were present (Demos et al., 2012). Indeed, as suggested above, music and social partners exert different influences (unidirectional versus bidirectional) on participants, possibly implemented by different mechanisms (temporal regularities versus common ground). When both music and a social partner are present, participants either have to integrate the two sets of information or choose just one set and ignore the other. In terms of word learning, if the establishment of a “common ground” is essential, then an improved word-learning rate should be observed in the social interaction condition, regardless of the fact that music also drives the learner’s attention toward the correct referent for new words. Instead, if this latter aspect is what drives word learning, then no difference should be observed between music and social interaction. However, another possible line of interpretation could be considered. Music may actually ease the cognitive dissonance arising from the stressful learning situation represented by the learning game. Cognitive dissonance is a well-known psychological phenomenon, describing a discomfort originated by holding conflicting cognitions (Festinger, 1957; Cooper, 2007; Harmon-Jones et al., 2009). Recent theories suggest that music may allow tolerating cognitive dissonance, hence facilitating the acquisition of new knowledge (Perlovsky, 2010, 2013a,b, 2014). If this were the case, then we should expect participants to perform better with music, independently of the presence of a partner (Masataka and Perlovsky, 2012; Cabanac et al., 2013; Perlovsky et al., 2013).

However, it may still be the case that neither music nor social interaction provides useful cues at all, as adult learners are cognitively equipped to learn new words without any additional help. In this scenario, music, and social interaction may, instead, interfere with learning, by increasing the cognitive load of the learning situation (Racette and Peretz, 2007; Moussard et al., 2012, 2014). To investigate these hypotheses, we manipulated the variability of the sentence context in which new words were embedded to obtain a “difficult” condition (that is, words were repeated in a different context so the word referent had to be identified *ex novo* at each occurrence) and an “easy” condition, in which task requirements were less demanding (that is, words were repeated in the same sentence context (sSC) so the referent was already known from previous presentations of the same word). In line with our previous results, we expected music and social cues to be maximally used in the “difficult” condition, but not used in the “easy” condition.

## Materials and Methods

### Participants

Eighty native German speakers (40 F, mean 24.86 ± 2.62 years) took part in the experiment. They were all recruited from a database from the Max-Planck Institute for Human Cognitive and Brain Sciences (Leipzig, Germany). All participants reported normal or corrected to normal vision, and none of them reported a history of hearing or neurological disorders. Right-handedness was assessed by the Edinburgh Handedness Inventory (Oldfield, 1971). An experimenter (LV, F, 28 years) was the partner in the social interaction conditions. The same experimenter participated in a previous pilot study with 68 German native speakers (34 F, mean 25.19 ± 2.88 years). This pilot study employed the same paradigm presented here, and was used to extract the time delays distribution used in the computer and music conditions in the current study to mimic the social condition. In both studies, all participants gave written informed consent and were paid for their participation. The experiment was conducted in accordance with the Declaration of Helsinki and approved by the Ethics Committee of the University of Leipzig.

### Materials and Apparatus

#### Visual Stimuli: Checkerboards and Pseudo-Words

**FIGURE 1:**
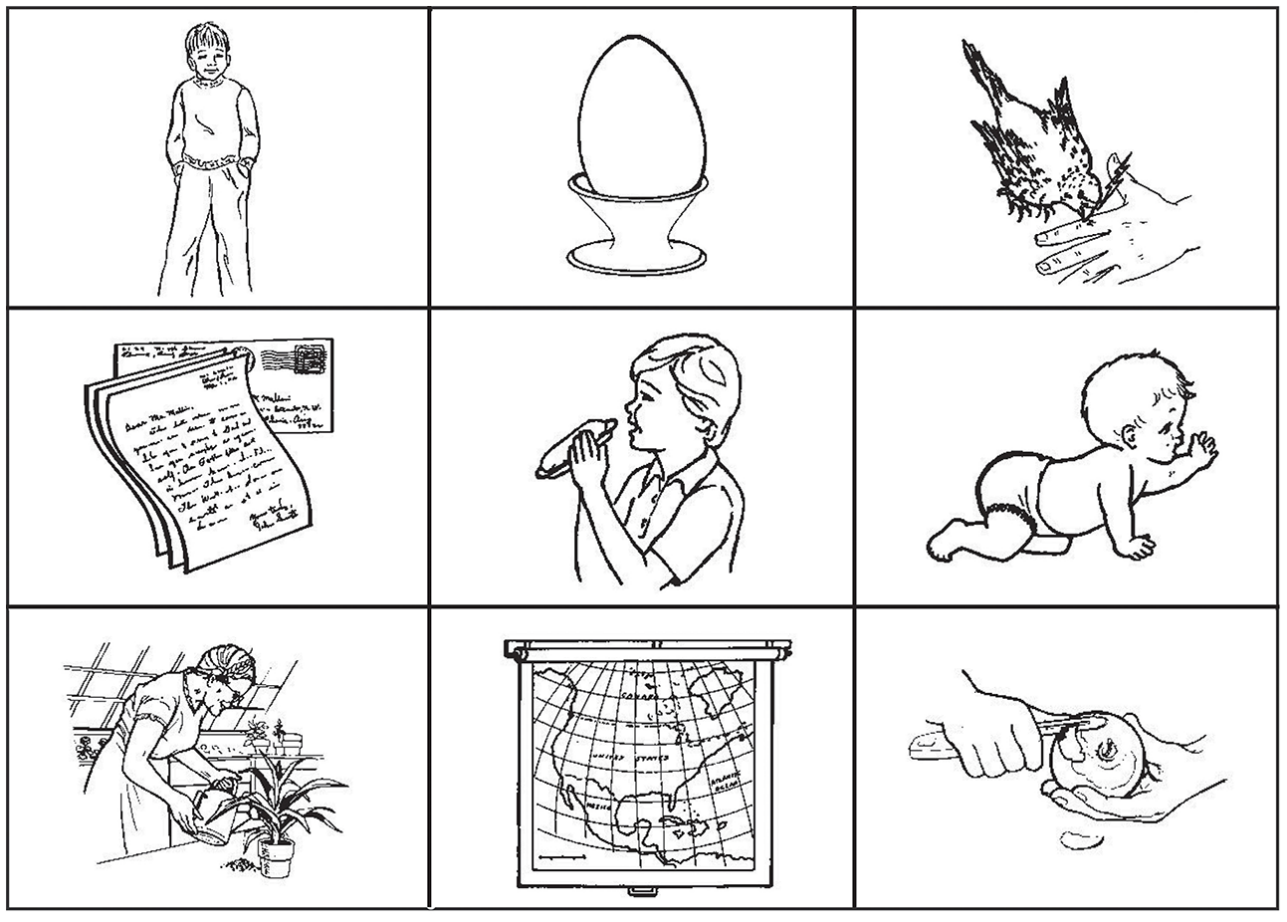
**Example of checkerboard used in the experiment**. The hidden sentence is in this example composed of the pictures representing a young boy, the act of eating, and the object egg. The sentence “The boy eats the egg” is the only plausible sentence that can be constructed within the given constraints. Elements depicted in the checkerboard are in the first row **(top)** from left to right: boy (noun), egg (noun), to pick (verb); second row **(middle)** from left to right: letter (noun), to eat (verb), baby (noun). Third row **(bottom)** from left to right: to water (verb), map (noun), to peel (verb). Images are reproduced from http://crl.ucsd.edu/experiments/ipnp/index.html

Visual stimuli consisted of 180 checkerboards (3 × 3) each containing nine images (330 × 245 pixels, 72 dpi) each centered in a different cell of the checkerboard. The images were black and white drawings representing objects, humans, animals, or actions selected from a validated database available online (Bates et al., 2003; Szekely et al., 2003, 2004, 2005; http://crl.ucsd.edu/experiments/ipnp/). A total of 49 images were employed, including 12 pictures representing humans, or animals (category: Subject), 17 representing actions (category: Verb), and 20 representing objects, humans, or animals (category: Object). All images represented single objects, humans or animals (**Figure 1**).

In each checkerboard, two nouns and an action were combined to form simple transitive German sentences (noun – transitive verb – target object; for example, “Der Junge isst das Ei,” “The boy eats the egg”). We defined the combination of subject (“Der Junge”) and verb (“isst”) as the “sentence context.” Images depicting elements of the sentence were represented in cells touching each other at least corner to corner. Given this constraint, only one object could be chosen to form a plausible German sentence. Subject and Verb pictures are presented to the participant, in each trial, in rapid succession; the way these pictures are selected depends upon the condition each participant is assigned to: In the social condition, the experimenter selects the two pictures, while in the computer and music conditions these are selected by the computer (see “2.4 Task and Experimental Procedure”). The delay between trial beginning (appearance of a checkerboard) and selection of the Subject picture varies from a repetition to the next, while the delay between Subject picture and Verb picture is kept approximately constant. The distribution of the delays was calculated based on a previous pilot study using the same paradigm and the same experimenter as the partner in the social condition; the delays used in the computer and music condition in the current study match the mean values of the experimenter-generated delays in each repetition of this pilot experiment. The experimenter-generated delays in the pilot study were highly correlated with the delays generated by the same experimenter in the current experiment (subject picture highlight: rs = 0.883, *p* = 0.002; verb picture highlight: rs = 0.950, *p* = 0.000). A summary of the delays in the pilot experiment and in the current experiment is presented in **Table 1**.

**Table 1:** The table summarizes the details of the timing sequences (mean and SD expressed in seconds) for the two main parts of each trial in the experiment (Subject picture highlight; time delay between Subject and Verb pictures highlight).

	Subject picture highlight onsets				Subject-verb pictures onsets delays			
	Experimenter				Experimenter			
Trial	Pilot study	Current study	Music	Computer	Pilot study	Current study	Music	Computer
1	4.650 ± 2.25	4.357 ± 1.06	4.642	4.650	0.652 ± 0.21	0.612 ± 0.06	0.658	0.500
2	3.424 ± 2.21	3.635 ± 0.57	3.429	3.424	0.514 ± 0.09	0.589 ± 0.08	0.658	0.500
3	3.110 ± 1.72	3.391 ± 0.49	3.117	3.110	0.498 ± 0.10	0.568 ± 0.10	0.658	0.500
4	2.972 ± 1.77	3.267 ± 0.57	2.972	2.972	0.521 ± 0.13	0.563 ± 0.12	0.658	0.500
5	3.040 ± 1.89	3.195 ± 0.63	3.038	3.040	0.491 ± 0.09	0.544 ± 0.12	0.658	0.500
6	2.774 ± 1.85	3.129 ± 0.51	2.775	2.774	0.489 ± 0.08	0.541 ± 0.13	0.658	0.500
7	2.936 ± 2.00	3.172 ± 0.55	2.936	2.936	0.480 ± 0.09	0.533 ± 0.13	0.658	0.500
8	3.000 ± 2.89	3.199 ± 0.62	3.000	3.000	0.479 ± 0.07	0.531 ± 0.14	0.658	0.500
9	2.634 ± 1.45	2.994 ± 0.53	2.637	2.634	0.463 ± 0.07	0.527 ± 0.14	0.658	0.500
	*r*_s_ = 0.883, *p* = 0.002				*r*_s_ = 0.950, *p* = 0.000			

*These time delays were not controlled by participants, but by the experimenter (S+ condition), music (M+ condition), or the computer (S- condition). The correlation between the experimenter-generated delays in the pilot study and in the current experiment is also reported. These time delays were not controlled by participants, but by the experimenter (S+ condition), music (M+ condition), or the computer (S- condition). The correlation between the experimenter-generated delays in the pilot study and in the current experiment is also reported*.

The six pictures not belonging to the target sentence were distractor images chosen from the initial image pool and were balanced between nouns (either animals, humans, or objects) and actions. None of these distractor images constituted a plausible object for the given sentence context. The checkerboards were further balanced for mean naming frequency of the depicted items and mean number of times each element of the target sentence (subject, verb, object) appeared in each cell. All possible dispositions for the three target images were employed a comparable number of times.

Images belonging to the category “objects” (*N* = 20), which were employed as targets for the sentence context, were each associated with a different pseudo-word. These stimuli were based on Italian word structure and were selected from a published set of disyllabic pseudo-words (Kotz et al., 2010). The selected pseudo-word sample (length range: minum 4, maxmum 6 l) was balanced for syllabic complexity, initial letter and final letter (“a” or “o”). We excluded words ending in “e” or “i” to avoid a possible confound with the Italian plural form, since all the pictures contained singular elements. Each pseudo-word and the associated target object could be presented a maximum of nine times during the learning phase of the experiment.

#### Auditory Stimuli: Melodies

Two original (i.e., unknown) melodies were created *ad hoc* by a music theorist (P.L.) to comply with our requirements. One melody was assigned to the “subject” of the sentence context, while the other melody was assigned to the “verb.” The melodies needed to parallel the role of the experimenter in the social condition as closely as possible. For this reason, the following criteria were applied: First, the length (duration) of the “subject melody” was adjusted to be comparable to the response times of the experimenter in the social interaction data previously collected. Thus, while the original melody was always the same, we ended up with nine different tempi, and progressively faster tempi were used from the first to the last repetition. The duration of the musical excerpts ranged from 2.64 to 4.64 s. To allow comparisons with the computer condition, the same durations were applied to jitter the stimuli in the silent condition. Second, a melody was created for the “verb” picture with a fixed duration of 658 ms. These time delays were comparable to the response times of the experimenter (which were extremely stable over the course of the experiment) to provide the “verb” picture and collected in a previous pilot study. A summary of the specific time delays is provided in **Table 1**. The rationale behind this choice not to extract the duration of these delays from a random distribution is based on the necessity to keep the structural and musical characteristics of the melodies intact. For the “verb delays” these were maintained at 658 ms, as this was close to the preferred tempo used by the experimenter in the pilot study, and confirmed in the current study. The “verb melodies” are slightly longer than the verb delays in the other two conditions (social and computer, 500 ms c.a.), as we found it virtually impossible to create a melody with a meaningful development lasting less than around 600 ms. Third, the choice of a single melody for each part was done to ensure comparability with both the social and computer conditions, characterized by a consistent “pacer” (same experimenter, same computer). Fourth, both melodies were simple with a clear development and a predictable ending point to ensure appropriate action from the participant when required.

### Experimental Design

We manipulated three factors: two levels of music (present, absent), two levels of social interaction (present, absent) and two levels of sentence context variability (same, different).

Music context and social interaction were both evaluated as between-subject factors. Every participant was semi-randomly assigned to one of four conditions: music and social interaction (M+, S+; *N* = 20, 10 F, mean age 24.40 ± 2.04 years), non-music and social interaction (M-, S+; *N* = 20, 10 F, mean age = 24.30 ± 2.23 years), music and non-social interaction (M+, S-; *N* = 20, 10 F, mean age 24.85 ± 3.12 years), and lastly non-music and non-social interaction (M-, S-; *N* = 20, mean age 25.90 ± 2.83 years). There was no age difference between the groups [all *p*s > 0.089]. The four groups were additionally balanced in terms of their musical background, defined in terms of years of musical practice prior to the participation in the study (mean number of years of instrument playing = 4.99 ± 6.37; mean number of years of singing and/or dancing = 1.94 ± 4.26; all *p*s > 0.210).

Half of the objects (*N* = 10) occurred repetitively within the sSC. For example, the image representing “the cow” was always the correct ending for the sSC “the wolf bites.” The other half of the objects (*N* = 10) was presented at each repetition within a different sentence context (dSC – different sentence context). For example, the image representing “the egg” could follow in sentence contexts such as “the woman cuts,” “the boy eats,” etc. The alternation between sSC and dSC checkerboards was randomized, as well as the order in which triads belonging to each of the two conditions were presented to each participant. Although each sentence was repeated nine times, the actual number of exposures to each pseudo-word was dependent on the number of correct responses given by each participant, as a pseudo-word was presented only in case of the correct object identification. The order of trial presentation was randomized for each participant.

### Task and Experimental Procedure

The experiment consisted of three parts: practice trials, learning phase, testing phase. Stimuli were presented using a desktop computer running Presentation 16.0 (Neurobehavioral Systems, Albany, NY, USA). Two standard wheel mice (Premium Optical Wheel Mouse, Logitech, Morges, Switzerland) were connected to the same Windows computer and used as response devices. Musical stimuli were presented via a stereo speaker system (LS21 2.1, Logitech, Morges, Switzerland). The task specifics are described below.

#### Practice Trials and Learning Phase

**FIGURE 2:**
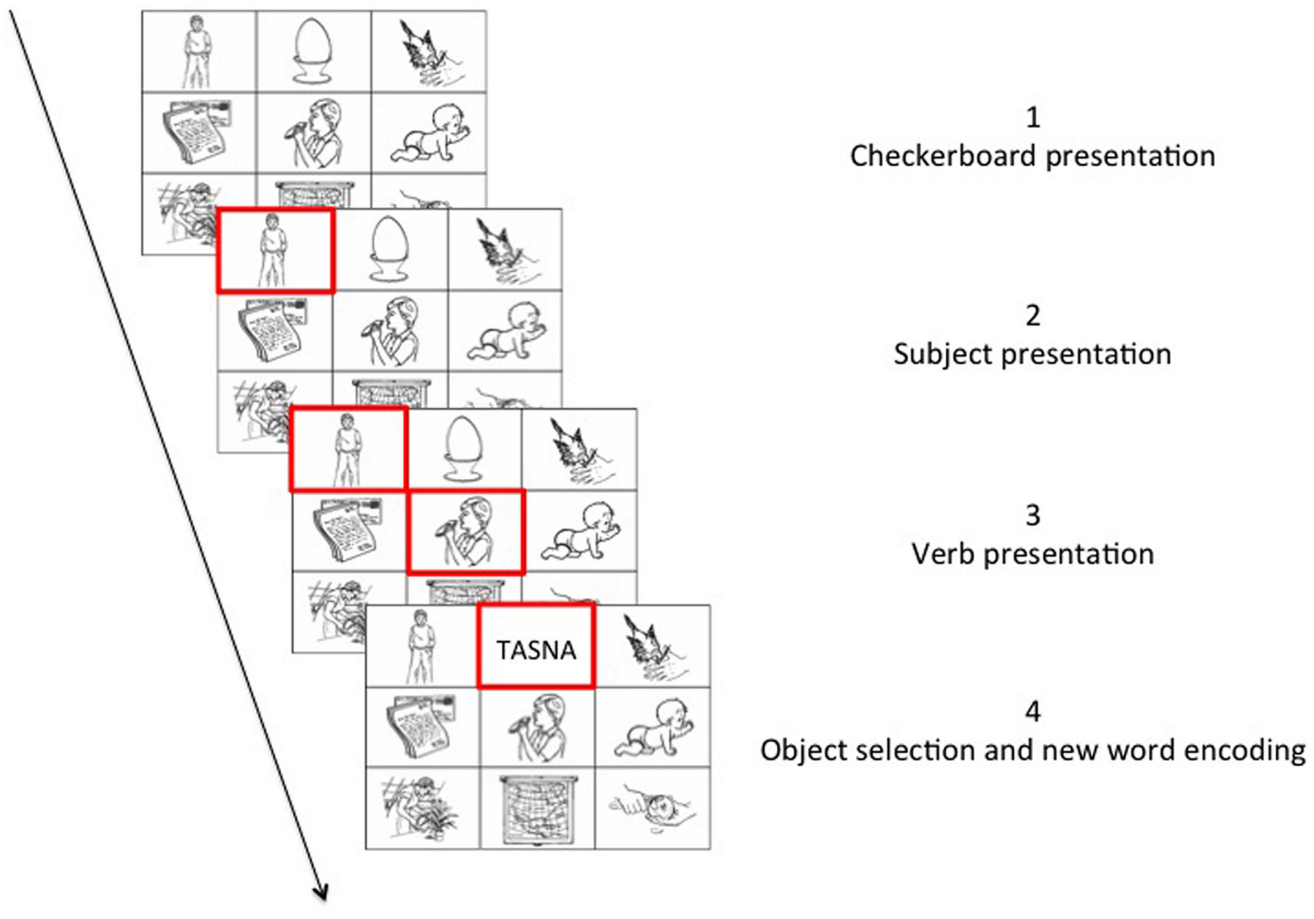
**Example of an experimental trial**.

Participants were first presented with detailed written instructions and performed a block of 10 practice trials to familiarize themselves with the task requirements. In all conditions, the task of the participant was to find the correct object for a given sentence context amongst the images on the checkerboards. Each trial began with the presentation of a fixation cross (500 ms), followed by a checkerboard containing nine images. In each checkerboard, a red frame appeared around the image representing the subject of the sentence context, followed by a second red frame around the image representing the verb of the sentence context. When both elements were marked with a red frame, the participant could give their answer by selecting an object fitting the sentence context from the remaining seven images on the checkerboard (**Figure 2**).

For participants assigned to the **social condition,** participant and experimenter were sitting side by side in front of the same computer screen; each member of the dyad was controlling a cursor on the screen through his/her own computer mouse. When the checkerboard containing the nine images appeared, the experimenter selected the subject and verb of the target sentence, by left clicking on them with the mouse in rapid succession. The experimenter tried not to adapt specifically to each participant’s behavior, but instead to keep a constant behavior consisting in a gradual speeding up over time. This decision was made to ensure the maximal compatibility with the non-social conditions, in which there was no adaptation to the participant’s behavior. For the participants assigned to the **non-social condition** (both M+ and M-) the sentence context was selected by a computer program. The delay between the appearance of the checkerboard and the marking of the “subject” of the sentence was randomly selected from a range comprised between 2.634 and 4.650 s; this range comprises nine different delays, each corresponding to the experimenter’s mean delay for each repetition in a previous pilot study (**Table 1**). These mean values were used in the current study to define, for each repetition, the delays between the appearances of the sentence context pictures. More specifically, in the **M**-**condition**, the red frame around the “subject” appeared with a variable stimulus onset asynchrony (range: 2.634–4.650 s; see **Table 1** for the repetition-specific delays); the red frame around the “verb” followed after 500 ms. In the **M+ condition**, a melody started playing when the checkerboard appeared; the “subject” red frame was highlighted at the end of the melody. The duration of the melodies was comparable to the stimulus onset asynchrony of the M- condition (range: 2.637–4.642 s; see **Table 1** for the repetition-specific delays); in this condition, the red frame around the “verb” followed after 658 ms. Importantly, when both music and social interaction were present (M+, S+ condition), the experimenter paid particular care to time her response to the offset of the musical stimulus. The selection of the delays between appearance of the checkerboard and appearance of the red frame around “subject” and “verb” pictures were based on the experimenter-generated delays in the social condition of a previous pilot study using the same paradigm and the same experimenter. These data are reported in **Table 1**, together with the experimenter’s time in this study and the delays used for the non-social conditions.

There was no time limit for participants to answer. In all conditions, if a correct answer was given, the selected image was substituted by a pseudo-word (black capital letters over white background, Arial, 40 pt.) providing the “Italian name” of the object. The pseudo-words remained on the screen for 1000 ms. If an incorrect response was given, no “Italian name” was displayed and the following trial began immediately.

After the training, participants performed the learning phase. The procedure of the learning phase was identical to the training phase. Hunderd and eighty trials (20 objects × 9 repetitions) were presented in total during the experiment. See Picture 2 for a graphical representation of a trial.

#### Testing Phase

At the end of the learning phase, a behavioral testing phase took place to evaluate whether pseudo-words presented during the learning phase had been mapped to the corresponding objects. In this task, participants were presented with novel sentence contexts (that is, combinations of pictures representing a subject and a verb that had not been seen together before), followed by three of the pseudo-words (“Italian words”) participants had learned during the learning phase. Participants were asked to select the “Italian word” that matched a given sentence context. All trials contained one correct and two incorrect options.

### Data Analysis

Statistical analyses of behavioral data were performed using MATLAB R2013a (The Mathworks Inc., Natick, MA, USA) and IBM SPSS Statistics 18 (IBM Corporation, New York, NY, USA). Behavioral data were first corrected for outliers. Trials with response times exceeding the mean ± 2 SDs were excluded from further analysis (mean rejected trials across participants = 4.32%).

Accuracy scores (proportion of correct responses in total), response times for correct responses and their SDs were calculated for each repetition of the object, for each participant and for each of the two conditions (sSC and dSC). For the learning phase, response times were calculated as the time delay between the appearance of the “verb” image and the participant’s answer. To evaluate the degree of temporal coordination of the participant during the learning phase, we used the following measures: First, SDs of response times were employed as an index of the stability of participants’ performance. We additionally used the coefficient of variation (CV) as an index of variability independent of response speed, to allow for a direct comparison between the different conditions. Further, we calculated the lag-0 and lag-1 cross correlation (cc) coefficients between the intra-trial-intervals produced by the participants (i.e., the time delay between highlight of the Verb picture and selection of the object picture) and those produced by the experimenter (S+ conditions) or computer (S- conditions; i.e., the time delay required to identify the subject of the sentence context). More specifically, the cc at lag-0 indicated how much the behavior of the participant in one trial was temporally related to the behavior of their partner (the experimenter/computer) in the same trial. Cross-correlations at lag-1 indicated whether the behavior of the experimenter/computer was related to the participant’s behavior in the following trial. There was no auto-correlation in the time series of the pacing signal, being either experimenter, computer or music (all *p*s > 0.066); furthermore, the same analyses conducted with a correction for auto-correlations yielded the same results as without correction. For this reason, the results here presented are based on cross-correlation indexes calculated without a correction for auto-correlations.

To account for the difference in the variability of trial presentation in the different conditions, we conducted separate ANCOVAs on the variables of interest using the SDs of the experimenter’s/computer’s response times as covariates during the learning phase. We did not use this covariate in the cross-correlation analyses as SDs account for the variability in the computer/experimenter RTs series, in which the correlation coefficients are calculated.

For the testing phase, response times were calculated as the time delay between the appearance of the three alternative pseudo-words and the participant’s response. Accuracy scores were defined as the proportion of correct responses out of the total number of responses. We used the number of exposures during the learning phase as a covariate. This number took into account the mean number of times pictures were repeated during the learning phase, ranging from a minimum of 0 (no correct responses) to a maximum of nine times (no errors).

When the assumption of sphericity was not met, a Greenhouse-Geisser correction was applied to the degrees of freedom. Two-tailed *t*-tests and simple effect analyses were employed to compare individual experimental conditions and to resolve interactions. We used an alpha level of 0.05 to ascertain significance for all statistical tests, and applied a Bonferroni correction in *post hoc* tests to control for multiple comparisons.

## Results

### Learning Phase

Participants responded with an average accuracy of 93% correct. A 2 × 2 × 3 repeated measures ANCOVA was conducted on accuracy scores with the between factors music context (M+ vs. M-) and social context (S+ vs. S-), the within factors sentence context (dSC vs. sSC) and repetition (nine repetitions), and SDs of presentation times (experimenter, computer) as covariates to account for differences in variability across conditions.

**FIGURE 3:**
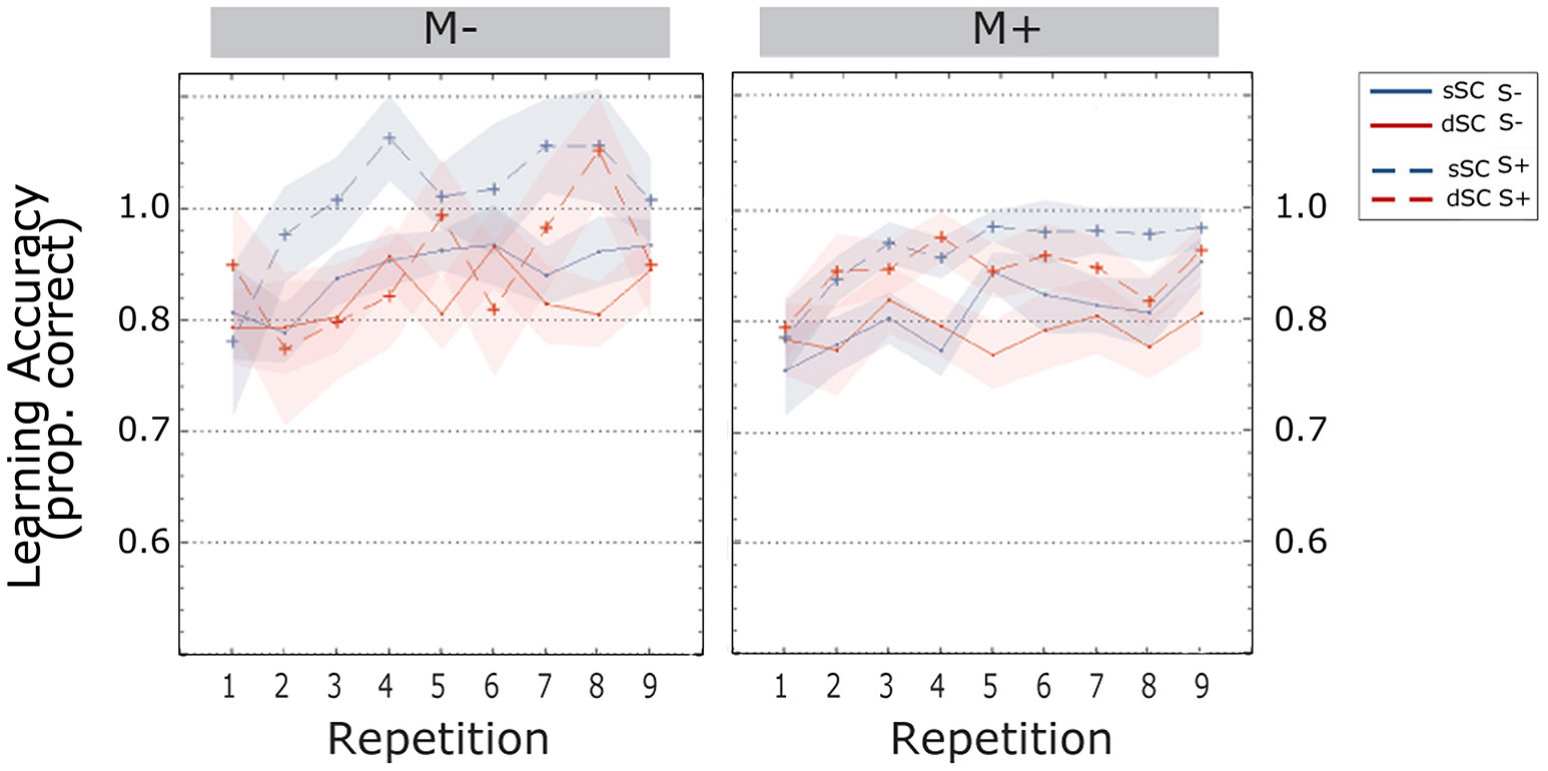
**Accuracy scores (estimated marginal means) during the learning phase, plotted as a function of item repetitions and controlled for time variability in sentence context presentation**. The area subtended by the shadows represents the standard error of the mean. M-, music context absent; M+, music context present; S-, non-social interaction; S+, social interaction; dSC, different sentence context; sSC, same sentence context.

Participants’ **accuracy** increased during the learning phase [linear trend, *F*(4.569, 333.552) = 5.798, *p* = 0.000, $\eta_{p}^{2}$ = 0.074]. Words encoded in sSC, (*M* = 0.954, SEM = 0.008) elicited higher accuracy than words encoded in different sentence contexts (dSC, *M* = 0.925, SEM = 0.009) [*F*(1,73) = 14.782, *p* = 0.000, $\eta_{p}^{2}$ = 0.168]. There were no other significant effects or interactions (all *p*s > 0.074; **Figure 3**).

**Response times** decreased over the course of the learning phase [linear trend, *F*(3.046, 219.321) = 34.332, *p* = 0.000, $\eta_{p}^{2}$ = 0.323]. Words encoded in different sentence contexts elicited slower response times (dSC, *M* = 3.339, SEM = 0.139) compared to words encoded in sSC, (M = 2.487, SEM = 0.107) [*F*(1,72) = 73.839, *p* = 0.000, $\eta_{p}^{2}$ = 0.506 ]. The interaction between repetitions and sentence context was significant: bonferroni corrected *post hoc* tests revealed no difference between sSC and dSC words at the first repetition (*p* = 0.863); however, response times for the two conditions started to differ already with the second repetition, with sSC being significantly faster than dSC during the entire learning phase (all *p*s < 0.001).

**FIGURE 4:**
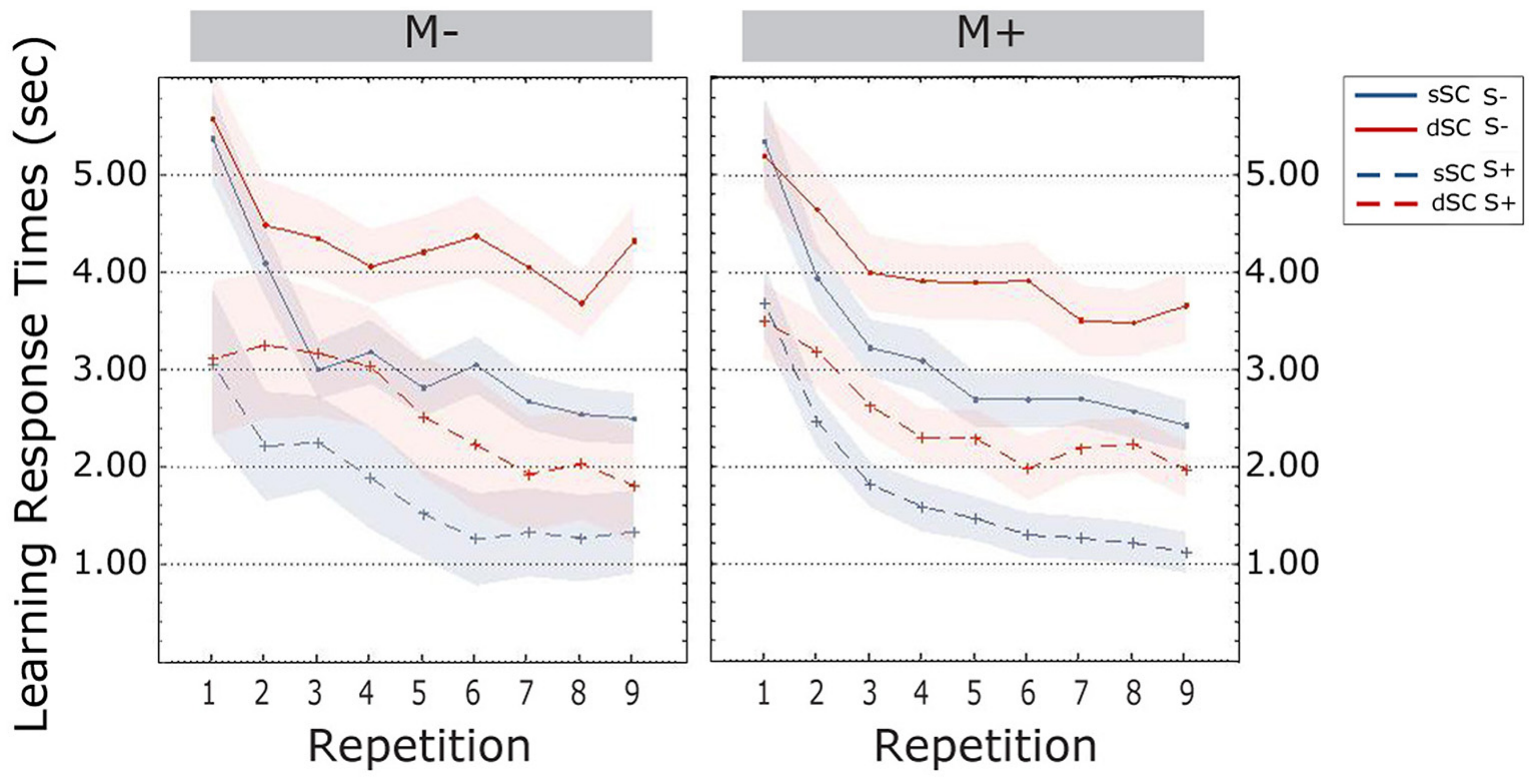
**Response times (estimated marginal means) during the learning phase, plotted as a function of item repetitions and controlled for time variability in sentence context presentation**. The area subtended by the shadows represents the standard error of the mean. M-, music context absent; M+, music context present; S-, non-social interaction; S+, social interaction; dSC, different sentence context; sSC, same sentence context.

Participants trained socially (S+, *M* = 2.325, SEM = 0.174) were significantly faster than participants trained non-socially (S-, *M* = 3.485, SEM = 0.174) [*F*(1,72) = 11.471, *p* = 0.001, $\eta_{p}^{2}$ = 0.137]. There were no other effects or significant interactions (all *p*s > 0.103; **Figure 4**).

**FIGURE 5:**
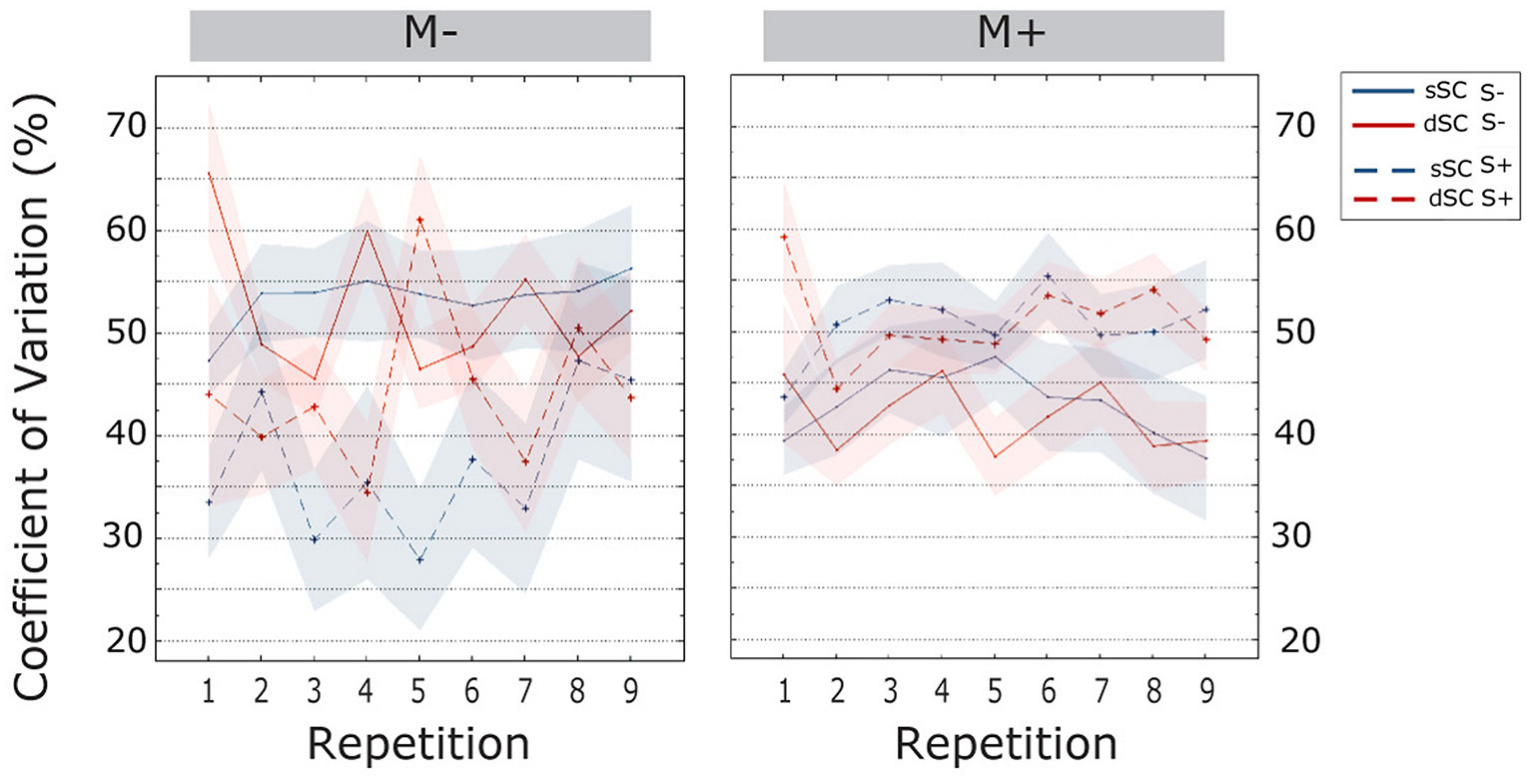
**Coefficient of variation (CV; estimated marginal means) during the learning phase, plotted as a function of item repetitions and controlled for time variability in sentence context presentation**. The area subtended by the shadows represents the standard error of the mean. M-, music context absent; M+, music context present; S-, non-social interaction; S+, social interaction; dSC, different sentence context; sSC, same sentence context.

The **CV** increased over the course of item repetitions [linear trend, *F*(6.355, 457.583) = 2.813, $\eta_{p}^{2}$ = 0.038]. Bonferroni corrected *post hoc* tests revealed that the CV was significantly lower in the first item repetition as compared to all subsequent repetitions (all *p*s < 0.033); further, in all repetitions except the third and seventh, the CV was lower than the last one (all *p*s < 0.038). Additionally, we observed an interaction between music context and social interaction [*F*(1,72) = 12.173, *p* = 0.000, $\eta_{p}^{2}$ = 0.145]. Therefore, a simple effect analysis was carried out. This analysis revealed that participants trained non-socially had significantly more stable performances when doing the task with music (*M*+, *M* = 0.373, SEM = 0.026) than without (M-, *M* = 0.478, SEM = 0.026) [*F*(1,72) = 13.681, *p* = 0.000, $\eta_{p}^{2}$ = 0.160]. In socially trained participants, we observed the opposite effect, though this was only marginally significant: participants performing the music task had significantly higher values of CV (*M* = 0.459, SEM = 0.020) as compared to participants doing the task without music (*M* = 0.357, SEM = 0.042) [*F*(1,72) = 3.825, *p* = 0.054, $\eta_{p}^{2}$ = 0.050]. There were no other significant effects or interactions (all *p*s > 0.099; **Figure 5**).

**FIGURE 6:**
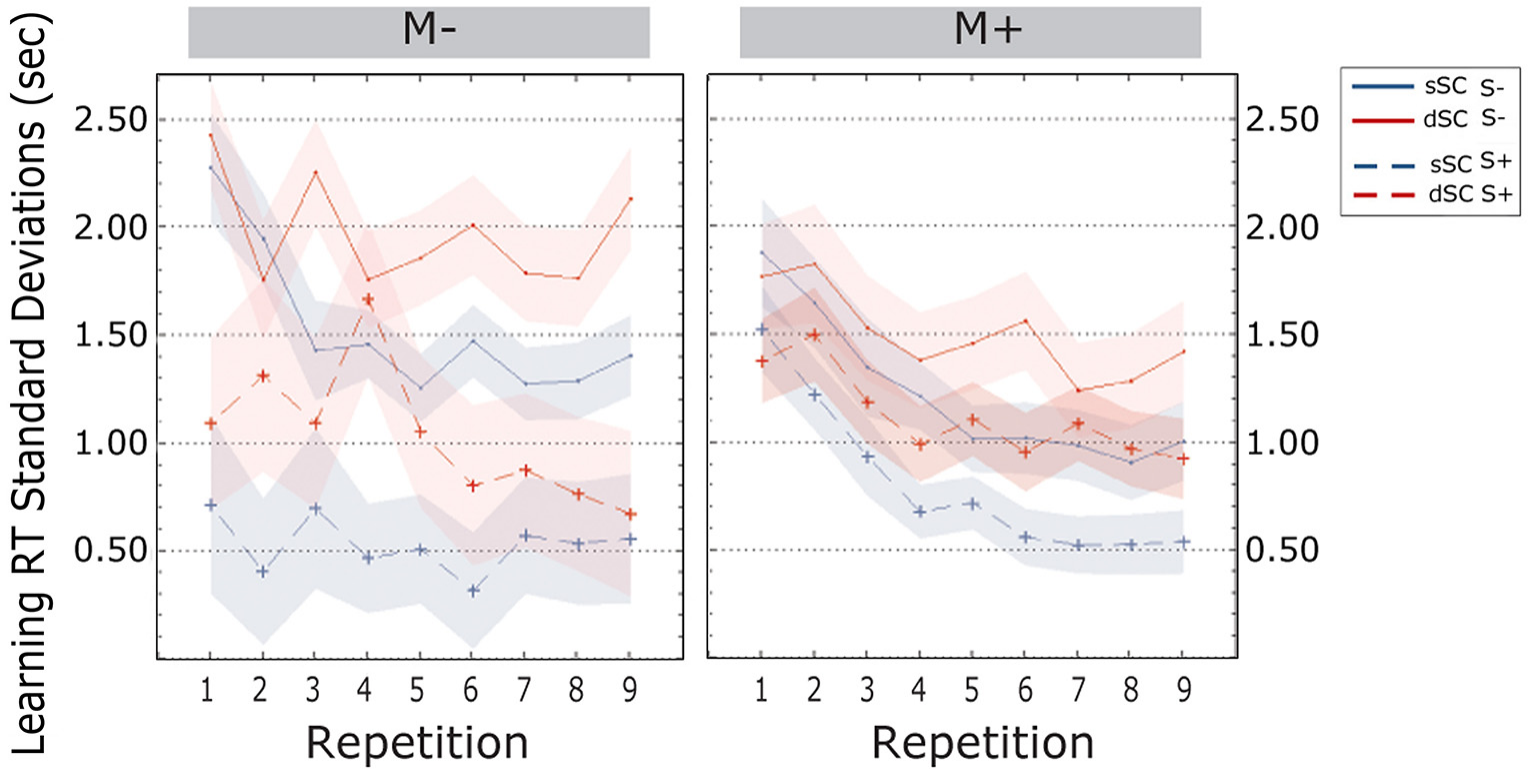
**Standard Deviations of response times (estimated marginal means) during the learning phase, plotted as a function of item repetitions and controlled for time variability in sentence context presentation**. The area subtended by the shadows represents the standard error of the mean. M-, music context absent; M+, music context present; S-, non-social interaction; S+, social interaction; dSC, different sentence context; sSC, same sentence context.

**Standard deviations of the response times** decreased over the course of the learning phase [linear trend, *F*(5.490, 395.256) = 3.625, *p* = 0.002, $\eta_{p}^{2}$ = 0.048]. Bonferroni corrected *post hoc* tests revealed that variability was significantly different between the first and the second item repetition (*p* = 0.000), between the second and the third (*p* = 0.019) and between the fourth and the fifth repetition (*p* = 0.020). There was no difference between the other transitions from one repetition to the next (all *p*s > 0.796). Further, SD for the responses to sSC words (*M* = 1.019, SEM = 0.051) were smaller than those to dSC word (*M* = 1.402, SEM = 0.071) [*F*(1,72) = 35.722, *p* = 0.000, $\eta_{p}^{2}$ = 0.332]. Additionally, participants trained in a social interactive context (S+, *M* = 0.869, SEM = 0.123) were less variable than participants trained non-socially (S-, *M* = 1.552, SEM = 0.130) [*F*(1,72) = 9.347, *p* = 0.000, $\eta_{p}^{2}$ = 0.115]. There were no further effects and no interactions (all *p*s > 0.113; **Figure 6**).

The **cross-correlations at lag-0** revealed a main effect of repetition [linear trend, *F*(1.897, 142.252) = 70.639, *p* = 0.000, $\eta_{p}^{2}$ = 0.485]; more specifically, Bonferroni corrected *post hoc* tests revealed a significant increase from one repetition to the next (all *p*s < 0.001) except for repetitions 4, 5, and 6 (all *p*s > 0.083).

The difference between the social and the non-social group was significant [*F*(1,75) = 8.044, *p* = 0.006, $\eta_{p}^{2}$ = 0.097]; indeed, participants trained socially had significantly higher lag-0 cc values (S+, *M* = 0.387, *SD* = 0.025) compared to participants trained non-socially (S-, *M* = 0.286, SEM = 0.025).

Further, the three-way interaction between sentence context, social interaction and music context reached significance [*F*(1,75) = 11.435, *p* = 0.001, $\eta_{p}^{2}$ = 0.132]. A follow-up simple effects analysis revealed that when participants were trained in a musical context, there were no differences if they were trained with a partner or without [*F*(1,75) = 1.260, *p* = 0.265, $\eta_{p}^{2}$ = 0.017], nor were there differences for sSC compared to dSC words [*F*(1,75) = 0.017, *p* = 0.897, $\eta_{p}^{2}$ = 0.000]. However, when learning without music, participants trained socially displayed significantly higher lag-0 correlations for dSC words compared to sSC words [dSC, *M* = 0.471, SEM = 0.044; sSC, *M* = 0.324, SEM = 0.043; *F*(1,75) = 9.323, *p* = 0.003, $\eta_{p}^{2}$ = 111]. There was no difference between sSC and dSC words for participants trained non-socially without music [*F*(1,75) = 0.291, *p* = 0.591, $\eta_{p}^{2}$ = 0.004].

**FIGURE 7:**
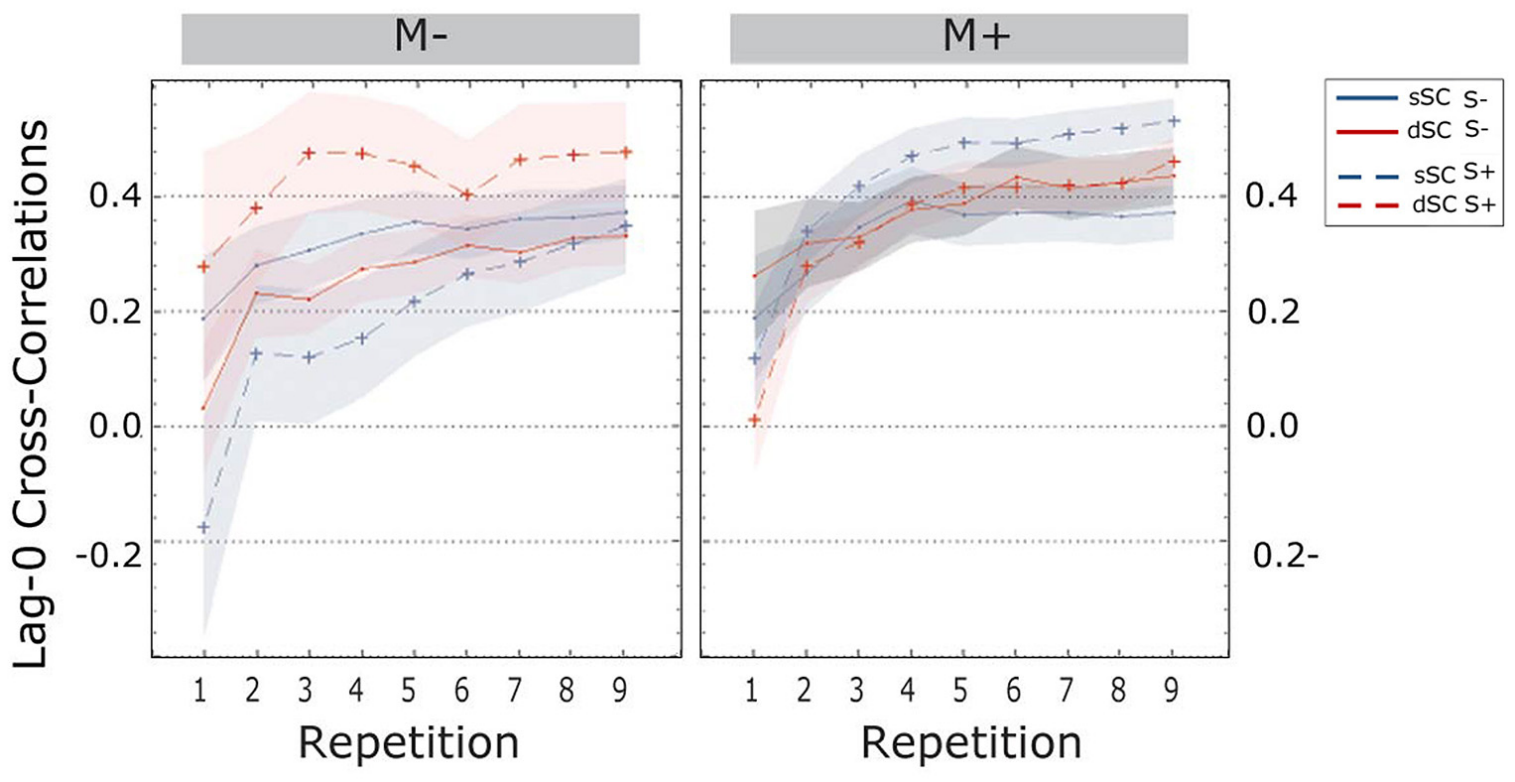
**Cross-correlations at lag-zero during the learning phase, plotted as a function of item repetitions**. The area subtended by the shadows represents the standard error of the mean. M-, music context absent; M+, music context present; S-, non-social interaction; S+, social interaction; dSC, different sentence context; sSC, same sentence context.

The three-way interaction between repetition, social interaction and music context was also significant [*F*(1.897,142.252) = 4.120, *p* = 0.020, $\eta_{p}^{2}$ = 0.052], therefore a simple effects analysis was carried out. This analysis revealed that when learning without music, participants in the S+ group had from the very beginning higher lag-0 cc (*M* = 0.245, SEM = 0.062) than participants trained non-socially (S-, *M* = 0.031, SEM = 0.061) [*F*(1,75) = 6.035, *p* = 0.016, $\eta_{p}^{2}$ = 0.074]. There was no difference when participants were trained with music at the first repetition [*F*(1,75) = 1.698, *p* = 0.196, $\eta_{p}^{2}$ = 0.022]. There was no difference between the two groups (S+ and S-) in either music condition (M+, M-) in repetitions 2, 3, and 4. Starting from the fifth repetition, participants learning without music became significantly more coordinated when trained with a social partner compared to a computer. This effect was then continuous until the end of the experiment (for all repetitions *p* < 0.025). The same significant difference was found in the musically trained group, but only starting from the second to last repetition (for repetitions 8 and 9 *p*s < 0.044). There were no other significant effects or interactions between factors (all *p*s > 0.120; **Figure 7**).

**FIGURE 8:**
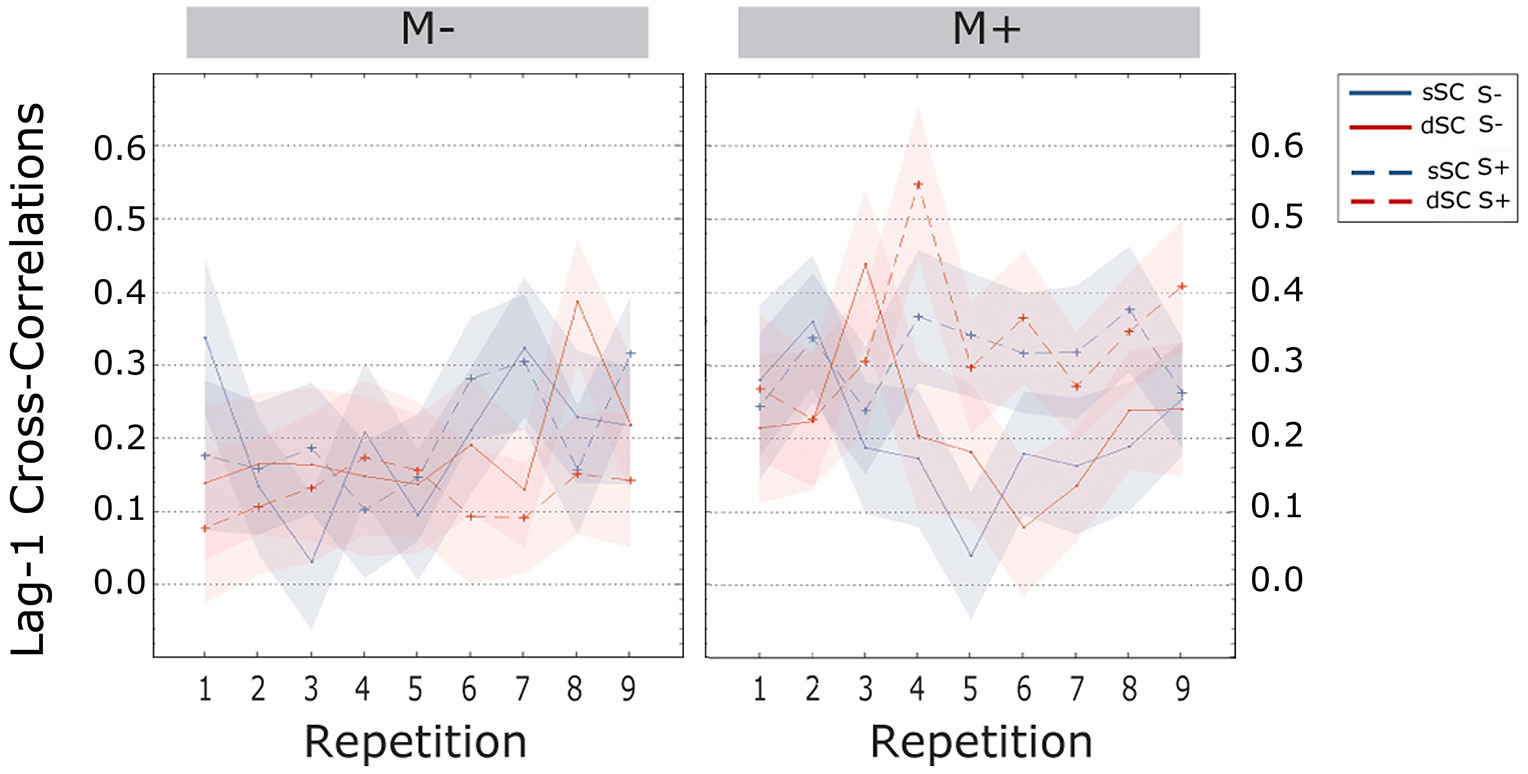
**Cross-correlations at lag-one during the learning phase, plotted as a function of item repetitions**. The area subtended by the shadows represents the SE of the mean. M-, music context absent; M+, music context present; S-, non-social interaction; S+, social interaction; dSC, different sentence context; sSC, same sentence context.

The **cross-correlations at lag-1** were significantly higher for participants trained with music (M+, *M* = 0.167, SEM = -017) than without (M-, *M* = 0.078, SEM = 0.017) [*F*(1,72) = 13.572, *p* = 0.000, $\eta_{p}^{2}$ = 0.159]. Further, the interaction between social interaction and music context was significant [*F*(1,72) = 8.676, *p* = 0.004, $\eta_{p}^{2}$ = 0.108], therefore a simple effects analysis was carried out. This analysis revealed no difference between participants trained socially or non-socially when learning without music [*F*(1,72) = 0.671, *p* = 0.415, $\eta_{p}^{2}$ = 0.009]. However, participants trained with music had significantly higher lag-1 correlations when playing with a partner (*M* = 0.224, SEM = 0.024) compared to a computer (*M* = 0.110, SEM = 0.024) [*F*(1,72) = 11.672, *p* = 0.000, $\eta_{p}^{2}$ = 0.137; **Figure 8**].

To summarize, learning effects emerged during the task with a progressive increase in accuracy and temporal coordination (lag-0 cc) and a decrease in response times. Overall, words encoded in a consistent sentence context were recognized faster and more accurately than words encoded in a different context. Participants trained socially were significantly faster, less variable (SDs) and more temporally coordinated (lag-0 cc) than participants trained non-socially. In the no-music condition, lag-0 cc were significantly higher for social participants exposed to dSC words. However, in the music condition no differences were observed. Variability independent of speed (CV) was lower for participants who trained non-socially with music than without; participants playing with an experimenter were instead more stable without music. Lag-1 cross-correlations were higher for participants trained with music, especially when playing the game with a partner.

### Testing Phase

Separate 2 × 2 × 2 ANCOVAs were conducted on accuracy scores and response times to evaluate the impact of the experimental manipulations (music context, M+ vs. M-; social context, S+ vs. S; sentence context, sSC vs. dSC) while accounting for the number of exposures to the pseudo-word during the learning phase.

**FIGURE 9:**
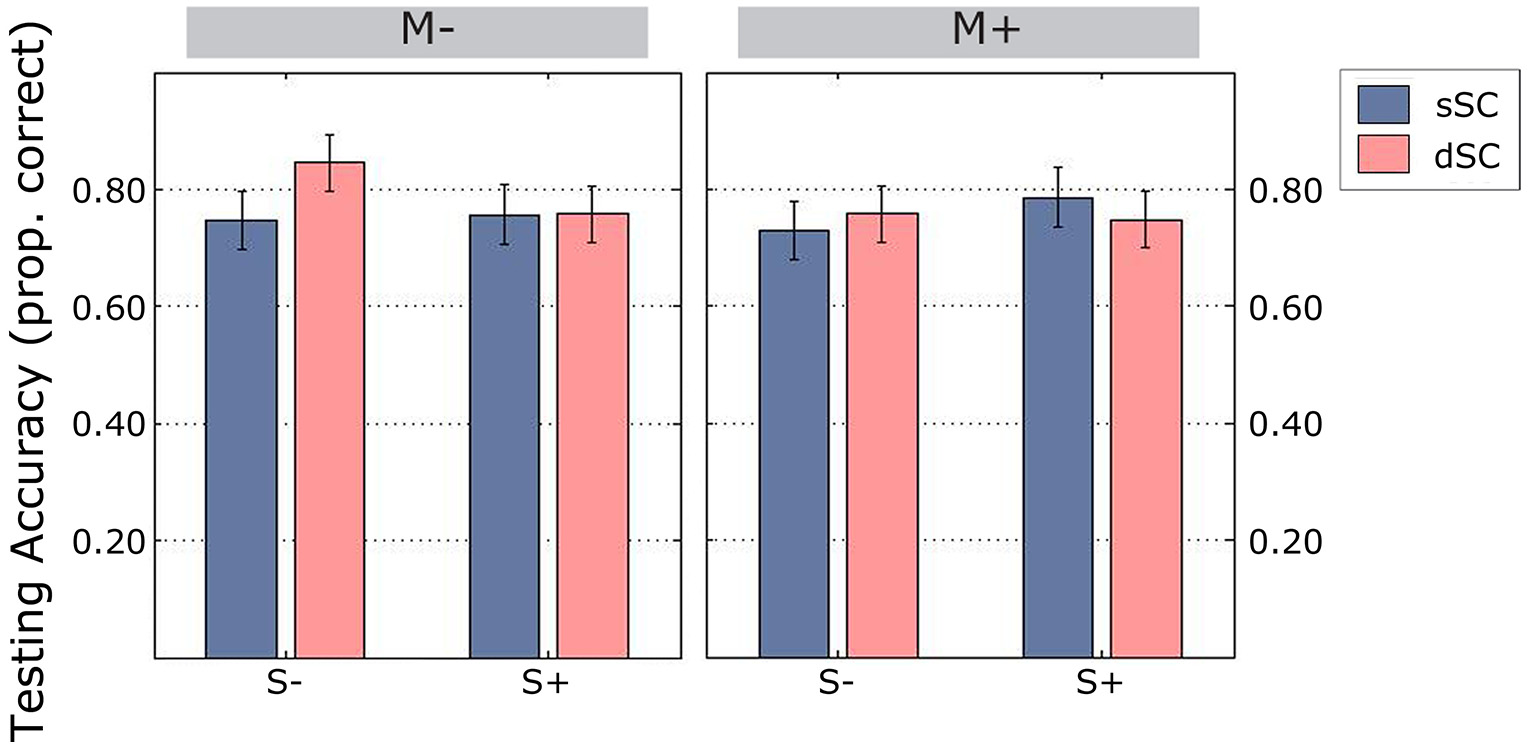
**Accuracy scores during the testing phase (estimated marginal means), controlled for mean number of repetitions during learning**. Vertical lines represent the standard error of the mean. M-, music context absent; M+, music context present; S-, non-social interaction; S+, social interaction; dSC, different sentence context; sSC, same sentence context.

Overall, participants performed at an accuracy level of 77%. We observed a significant interaction between sentence context and social interaction [*F*(1,75) = 4.605, *p* = 0.035, $\eta_{p}^{2}$ = 0.058], therefore a simple effects analysis was carried out. This showed that there was no difference between sSC and dSC words in the group of participants trained socially [*F*(1,75) = 0.465, *p* = 0.497, $\eta_{p}^{2}$ = 0.006]. However, participants trained in the S- condition correctly identified more dSC (*M* = 0.801, *SD* = 0.209) than sSC (*M* = 0.739, *SD* = 0.233) words, [*F*(1,75) = 5.536, *p* = 0.021, $\eta_{p}^{2}$ = 0.069; **Figure 9**]. There were no other significant interactions (all *p*s > 0.151) and no significant main effects (all *p*s > 0.204).

Response times during the testing were not significantly different between conditions when controlling for mean repetitions during the learning phase (all *p*s > 0.193). In summary, during the testing phase, participants trained non-socially remembered more words originally encoded in different sentence contexts.

## Discussion

The aim of the current study was to investigate the impact of music and social interaction on word learning in adult speakers. Both types of context have been hypothesized to enhance attention toward relevant information in the learning environment (that is, the referent for a new word), by exerting a unidirectional (music) or bidirectional (social interaction) temporal influence on the learner. To address whether this difference impacts the way new words are learned, we implemented a game set-up, in which participants learned new words embedded in sentence contexts with different degrees of variability. Our results show that participants were significantly faster, less variable and more temporally coordinated when learning with a partner, than when participants were trained non-socially. When learning without music, participants trained socially displayed better coordination during variable (“difficult”) context trials compared to consistent (“easy”) context trials. However, coordination with music, especially when playing with a partner, tended to be delayed from one trial to the next. Variability, when accounting for differences in response times, was lower for participants learning non-socially in the music condition. Finally, in the testing phase, participants trained non-socially remembered more words originally presented in different sentence contexts, although words repeated in a consistent context represented an easier condition (confirmed by the faster reaction times and higher accuracy in this condition during learning). While these results are in line with previous evidence of spontaneous temporal coordination during social interaction, they also provide a significant advance for research on communication and word learning in adults; indeed, they suggest that not only are adult learners influenced by the presence of a social partner, but also that this influence is different from the one exerted by other sources, such as music.

The results presented here support previous literature showing that temporal coordination spontaneously emerges during social exchanges (Richardson et al., 2007; Demos et al., 2012; Yun et al., 2012). Indeed, participants performing the task with a social partner were faster, less variable, and more temporally coordinated with the experimenter than participants performing the task with a computer. Temporal coordination with music had a weaker effect as compared to social interaction, as participants coordinated their behavior with the music stimuli immediately preceding the one they were listening to. These results can be interpreted within the framework of coupled oscillators (Wilson and Wilson, 2005; Dumas et al., 2010; Demos et al., 2012). In brief, this hypothesis proposes that since human movements tend to be rhythmic, two people performing a joint task are not dissimilar from other systems displaying periodic variations in time. As a consequence, interacting human dyads respond to the same dynamics as other oscillators; that is, they reciprocally influence each other in order to reach an equilibrium (Richardson et al., 2007; see also Kelso, 1997). Music, on the other hand, represents a unidirectional influence. In the present study, participants coordinated with the temporal regularities of the music, but the lack of reciprocal adaptation reduced the extent of the coordination. While this result seems in contrast with evidence that has consistently shown a strong effect of music on temporal coordination, it must be noted that, in most previous studies investigating sensori-motor synchronization, participants have been explicitly required to coordinate with continuously playing sequences (Pecenka et al., 2013; Repp and Su, 2013). Instead, we wanted to exploit *spontaneous* coordination with a *temporally defined* musical excerpt (that is, the musical sequence was finite for each trial and participants were required to take action at the end of the sequence, not during it), in order to maximize the music’s potential to drive the learner’s attention to a specific point in time. Results from the condition in which both music and a partner were present at the same time further corroborate this interpretation: music and social interaction may be responsible for different forms of coordination, due, in turn, to different underlying mechanisms. Indeed, participants learning socially are significantly more variable in their responses when learning with music, while the opposite is true for participants learning alone (we observed less variable performances with music). This increased behavioral uncertainty likely depends on the different influences stemming from the two sources. While without music there is only one source of information (the experimenter), music introduces a second set of coordinative cues; since the two sources exert different influences (unidirectional versus bidirectional), there may be uncertainty as to what one should coordinate to. In turn, this uncertainty is behaviorally reflected in increased response variability. However, this uncertainty is likely transient; increased coordination with the experimenter (compared to the computer) when music was present, emerged only toward the end of the learning phase, much later than without music. Furthermore, this coordination with music was maximal between responses in one trial and the music excerpt of the preceding trial, but not with the music in the trial participants were responding to; in other words, participants’ tended to have response patterns which reflected the duration of the previous musical stimulus, but not the one they were answering to. Another explanation for these results may be that in the current task, music was employed concurrently with another high-level cognitive task (identifying a sentence on the checkerboard). Despite the relative simplicity of the musical stimuli that we employed, the combination of music and task demands may have been too challenging for music to actually facilitate the performance (Kang and Williamson, 2013). However, the lack of difference in response accuracy or reaction times in the music and non-music conditions tends to rule out this possibility. Further discarding this possibility, music has been proven to ease the cognitive dissonance arising from stressful testing conditions (Masataka and Perlovsky, 2012; Cabanac et al., 2013; Perlovsky et al., 2013). In a recent series of studies, Perlovsky et al. (2013) showed that students performing an academic test obtained better results when listening to pleasant music as compared to a silent environment (Perlovsky et al., 2013), and, more generally, that students who choose a music course in their curriculum tended to achieve better grades (Cabanac et al., 2013). The authors suggest that music may help to mitigate the cognitive dissonance arising in these stressful contexts by virtue of its emotional value, thus facilitating the accumulation of knowledge (Perlovsky, 2010, 2012, 2013a,b, 2014). While the current results do not support this hypothesis, the different outcomes may arise from the use of repetitive, short, and novel musical sequences in the current experiment, while Masataka and Perlovsky (2012) and Perlovsky et al. (2013) employed Mozart musical pieces playing as a background during the academic tests.

So far, the results of the learning phase suggest that temporal coordination to music and a social partner have different characteristics, possibly reflecting different underlying mechanisms. But what are the implications for word learning? Both music and social interaction have been claimed to facilitate word learning and memory (Rainey and Larsen, 2002; De Groot, 2006; Jeong et al., 2010; Ferreri et al., 2013, 2014; Verga and Kotz, 2013; Ludke et al., 2014); several accounts explain this effect as the result of the easiness – for these stimuli – to allow predictions on the upcoming events and allocate one’s attention accordingly (Gogate et al., 2000; Lagarde and Kelso, 2006; Rolf et al., 2009; Rader and Zukow-Goldring, 2012; Schmidt-Kassow et al., 2013). The data presented here, however, suggest that the behavioral adjustments participants make may be based on different kinds of predictions. In the case of music, predictions are based on the temporal structure of the stimulus (unidirectional influence), while in the case of a partner they rely on the ability to infer the other person’s intention (bidirectional influence; Frith and Frith, 2006, 2012). This allows the creation of a “psychological common ground,” in which the transmission of information is facilitated (Tomasello, 2000; Csibra and Gergely, 2009). In this shared psychological space, the increased temporal coordination observed in this study may reflect a strategy that a knowledgeable partner uses to direct the learner’s attention toward the correct referent for a new verbal label (Pereira et al., 2008). Thus, the attention of the learner is focused on the target referent, consequently facilitating the mapping of a new word onto its meaning. This account predicts that temporal coordination with a knowledgeable partner should be better when the learner does not know *a priori* where the target referent may occur. In this situation, the adult learner is similar to a child learning its first words and faced with a constantly changing environment, in which multiple referents are present. Our results show that, indeed, temporal coordination with the experimenter was higher in this contextual condition. However, no differences were found between music and non-music conditions in relation to the variability of the context that words were embedded in. An interpretation of this result is that a shared psychological space – behaviorally reflected in the temporal coordination with a partner – is used by adult learners to identify a referent for a new word, when it cannot be extracted by the context of the word presentation alone. That is, participants “disengage” from social interaction if they can identify a referent by themselves. Instead, the presence of music overrules contextual diversity, as participants maintain the same pattern of coordination independently from the characteristics of a word presentation. This result is somehow in-between the two opposing accounts of the adult learner, one suggesting that adults are entirely self-sufficient learners (Pickering and Garrod, 2004; Stephens et al., 2010) and the other suggesting a critical role for others in shaping cognitive activity (Schilbach et al., 2013; Ciaramidaro et al., 2014; Schilbach, 2014; Sebastiani et al., 2014); indeed, these results suggest that the presence of another person is used *when needed*. While our results indeed confirm that music and social interaction may drive attention in different ways, the question remains open as to which strategy may be more relevant to successfully learning new words. An important implication of these results concerns situations in which music and social interaction are present at the same time, especially for tasks requiring coordination to either one of the two stimuli. Music therapy represents an important example of this situation. In addition to its positive effect on mood and arousal (Sarkamo et al., 2008), music is often employed to provide the patient with a temporal structure to facilitate her/his performance (Stahl et al., 2013, 2011), while at the same time a therapist needs to be present with the patient (Norton et al., 2009). The competition observed in this study between music and a social partner as coordinative tools suggest that their respective roles should be further investigated in these types of settings.

Quite surprisingly, during the testing phase, participants that were trained non-socially correctly identified more words when they had originally been presented in variable sentence contexts (as opposed to consistent sentence contexts), while no differences were observed either in the social group or in the music groups. In general, an advantage of words repeated at each occurrence in a different context is to be expected, as every time the same word is encountered in a different context, different contextual cues accumulate and enrich the representation of the target referent and its association with the new word (Verkoeijen et al., 2004; Adelman et al., 2006; Lohnas et al., 2011). Nevertheless, according to the hypothesis that a social partner and music may help the learner in directing attention toward the target (although through different mechanisms), an advantage of music and social interaction over simple computer learning should be expected. We provide two possible explanations for these results: first, while learning new words from a computer interface and testing participants with a computer interface is consistent, participants who learned with social interaction and/or with music may have been disadvantaged as they experienced a contextual inconsistency between the learning and the testing phase. Indeed, consistency between learning and testing environments has been suggested to facilitate recall (Godden and Baddeley, 1975; Polyn et al., 2009). This hypothesis, known as the “transfer appropriate processing” theory, states that the strength of a memory trace (that is, the ease of its retrieval) depends on the type of encoding compared to the type of retrieval (Stein, 1978; Tulving, 1979); if the form of encoding is congruent with the type of testing, retrieval is facilitated. In this study, the social and the music group faced an incongruity between the learning phase and the retrieval phase, which was always conducted by participants alone and without music. Instead, the non-social groups were exposed to the same type of encoding and testing (both alone and without music). An explanation based on incongruence between the type of encoding and the type of testing has been suggested in other learning studies; for example, Peterson and Thaut (Peterson and Thaut, 2007) found no behavioral advantage for sung compared to spoken word lists in an explicit verbal learning task, in which words were sung during learning and spoken during the recall phase. However, a behavioral advantage for sung stimuli emerged when participants were instructed to sing back during the recall phase (Thaut et al., 2008, 2014; for a review see Ferreri and Verga, under review). Further investigation is required to clarify this aspect, by testing participants not only in the non-social, silent condition, but also in the same condition they were trained in. Results in this direction would have important implications in terms of the extent to which acquired knowledge may be generalized to different contexts. If the context of word acquisition needs to be the same at retrieval, this would have little facilitation in some conditions. As an example, if the same music a word was learned with needs to be present every time the new word is used, it would not be a particularly helpful learning aid. The case of social interaction somehow represents an exception, as words are often (although not always) learned with someone (for example, in the case of first language learning) and used to communicate with someone. In this learning situation, the context of a word acquisition is often the same as the context of use. Hence, in this condition, results favoring the transfer appropriate theory would not be as problematic as for the case of music: Not always, indeed, it is possible to play the same music a new word was encoded in when retrieving the word in the context of use. However, another possible explanation for the observed results could be considered: The longer reaction times observed during learning in the non-social, silent condition may represent a strategy employed by the participant to look more in depth at the checkerboard. Using this strategy, they would have had more time to analyze the sentence context and find the correct target object. If this was the case, this would mean that – in the current task – the optimal strategy would be *not to* coordinate with the sentence context. While this is a possible explanation for the observed results, it may not be the most plausible: indeed, despite the differences in reaction times between conditions, there was no difference at the accuracy level; if the longer time spent observing the checkerboard provided an advantage, then we would also expect more correct responses in this condition. More importantly, however, the presentation of the “new name” of the target object (i.e., the word to be learned) was presented to the participants only after the selection of the correct object – independently of the time spent looking at the checkerboards – and remained on the screen for an equal amount of time in all conditions.

Second, our testing phase took place immediately after the learning phase, and therefore we did not consider consolidation effects that have been deemed important for word learning in both children and adults (for example Henderson et al., 2013). Social context has been proven to significantly bias the formation of new memories. For example, in a study by Straube et al. (2010), participants watched video clips of an actor speaking to them directly or to a third person. Source memory (the memory of the context a sentence was heard in) was significantly biased by social interaction, as participants tended to report that the actor was talking to them even if he was not. In our experiment, the testing phase took place immediately after encoding and it did not provide information concerning possible long-term mnemonic effects, which critically depend upon consolidation processes (Walker and Stickgold, 2004). As the efficacy of consolidation depends on several factors, among which sleep seems to play a particularly pivotal role (Siegel, 2001; Stickgold, 2005; Diekelmann and Born, 2007; Atherton et al., 2014; Lewis, 2014), a possible way to test long-term effects of social interaction may be by testing retrieval at delayed time points after the learning phase has taken place. These delayed time points would include short intervals within an hour, as well as longer intervals (days or weeks). This way, it would be possible to obtain a map of the long-term and consolidation effects as a function of the time passed since the learning took place.

## Conclusion

The current study aimed at investigating the respective roles of music and social interaction as possible facilitators of word learning in healthy adult speakers. We found that social interaction, more than music, improves temporal coordination in a verbal learning task. Further, music, and social interaction provide different types of influence (unidirectional versus bidirectional) that do not combine together easily, as the presence of social interaction and music at the same time hinders coordination. Crucially, the quality of coordination with the human partner (but not with music) is intertwined with the attentional demands of the task at hand; coordination is higher when it is difficult to find a new word’s referent. Taken together, these results support the notion that music elicits a different form of temporal coordination from the one observed in interacting dyads, whose behavior is compatible with coupled oscillators. This result has important implications for situations in which music and social interaction are present at the same time, such as many forms of music therapy. Although different, these forms of coordination equally impact word learning, as seen in the testing phase immediately following the task. This result calls for further studies to elucidate the extent to which the context of learning and its modulating factors (such as cognitive dissonance) influence performance during retrieval and how they may be influenced by consolidation processes.

## Conflict of Interest Statement

The authors declare that the research was conducted in the absence of any commercial or financial relationships that could be construed as a potential conflict of interest.

## Footnotes

1 For consistency, the term “temporal coordination” is used in this paper to describe the establishment of temporal dynamics between participants and music or a social partner. It must be pointed out that the same phenomena are described by other authors as *synchronization* (for example Yun et al., 2012), *entrainment* (for example Knoblich and Sebanz, 2008), mutual *adaptation* (for example Konvalinka et al., 2010) or with the more general term *coupling* (for example Demos et al., 2012).
